# A cross-sectional study of women’s autonomy and modern contraception use in Zambia

**DOI:** 10.1186/s12905-022-02101-5

**Published:** 2022-12-27

**Authors:** Abigail Mangimela-Mulundano, Kirsten I. Black, Kate Cheney

**Affiliations:** Faculty of Medicine and Health, The University of Sydney, Sydney, NSW Australia

**Keywords:** Maternal and child health, Women’s autonomy, Modern contraception, Zambia, Demographic health survey

## Abstract

**Background:**

Modern contraceptive use effectively prevents unwanted pregnancies, promoting maternal and child health and improving the socio-economic well-being of women and their families. Women’s autonomy has been shown to increase the uptake of modern contraception use. This research aimed to investigate the relationship between measures of women’s autonomy and modern contraception use among partnered women in Zambia.

**Methods:**

This cross-sectional survey study used data from the health census, the 2018 Zambia Demographic Health Survey. We measured women’s autonomy using three indices: women’s participation in decision-making, women’s attitude towards wife-beating and women’s household status. Information from 6727 women in a relationship, not pregnant, not planning pregnancy and aged between 15 and 49 years old were analyzed using descriptive statistics and adjusted odds ratios (AOR).

**Results:**

The mean age of respondents was 32 years. Most women lived in rural areas (65%), and 81% were protestant. Current modern contraception use among partnered women was 8.8%. Women’s autonomy was significantly associated with modern contraception use. Women with moderate autonomy (AOR = 1.054, *P* value = 0.004, 95% CI 1.048–1.312) and high autonomy (AOR = 1.031, *P *value = 0.001, 95% CI 1.013–1.562) had higher odds of using modern contraception compared to those with low autonomy. Other factors related to modern contraception use included a higher level of education (AOR = 1.181, *P* value = 0.012, 95% CI 1.091–1.783), increased wealth index (AOR = 1.230, *P* value = 0.006, 95% CI 1.105–1.766) and age, 15–24 (AOR = 1.266, *P *value = 0.007, 95% CI 1.182–2.113,) and 25–34 (AOR = 1.163, *P *value = 0.002, 95% CI 1.052–1.273).

**Conclusion:**

This study argues that increasing women’s assertiveness to make independent decisions within the household is cardinal to enhancing the uptake of modern contraception in Zambia and other low-and-middle-income countries. Governments and other stakeholders must therefore consider rolling out programs to boost women’s autonomy, which in turn would support gender equality and reproductive health.

## Background

The promotion of sexual and reproductive health including access to effective modern contraceptive methods, is an effective strategy for preventing unwanted pregnancies which can reduce maternal morbidity and mortality including from unsafe abortion [1, 2]. Importantly, prevention of unwanted pregnancies reduces the socioeconomic and educational constraints that come with childbirth [1, 2].

Modern contraception use is defined as the use of a range of reliable methods that effectively prevent pregnancy [3–5]. The definition includes female and male sterilization, intra-uterine devices (IUD), implants, injectables, oral contraceptive pills, vaginal rings, hormonal patches, male and female condoms, vaginal barrier methods (including the diaphragm, cervical cap and spermicidal foam, jelly, cream, and sponge), lactational amenorrhea method (LAM) and emergency contraception [4, 5]. This range of family planning options means that women can choose the method that fits their circumstances [5, 6]. In being effective in preventing pregnancy, modern contraception offers women the right to decide the number, timing and spacing of any children, if desired and for greater involvement in other productive activities such as education and employment [5, 7, 8].

In Zambia, the contraception prevalence rate increased from 33% in 2012 to 50% in 2018 and there is now almost universal knowledge of modern contraception among partnered women (99%) [4, 5]. However, actual use of modern methods in this population is still low at 48% and below the target of 58% [4]. In other similar settings, this low uptake of contraception has been attributed to a lack of autonomy in contraceptive decision making. Previous studies in Zambia have explored modern contraception use in association with contraception decision-making and socio-demographic factors [5–9]. The link between women’s autonomy defined as the capacity for one to make knowledgeable, independent choices free from external influences and household decision-making among married women in Zambia has been explored in a study using 2013–2014 Zambian Demographic Health Survey (ZDHS). The study found women with greater autonomy were more likely to participate in decision-making that involved daily household purchases [10–12].

Autonomy also enhances women’s assertiveness in dealing with sexual and reproductive health choices, but there are limited data in terms of its research and reporting [13, 14]. Autonomy is challenging to measure and, as in the DHS, often relies on composite indices. that present a summery score [11–14]. This study sought, for the first time, to explore the association between Zambian women's autonomy (from composite of three indices of women’s participation in decision-making, women’s attitude towards wife beating (domestic violence) and household status measured by property ownership) and modern contraception use [11–13].

## Methods

This cross-sectional survey study used secondary data from the 2018 ZDHS. The details of how this countrywide survey was conducted are described in the ZDHS report [4]. Partnered women (married or cohabiting), not currently pregnant, and not planning for pregnancy before two years were included in the analysis. Written permission was obtained from the DHS program before the start of the project. Confirmation was given to access the datasets on 12 May 2021. The letter for approval is attached and available for review. Procedures for ethical approval are as stated by the DHS website available at: https://dhsprogram.com/data/

### Dependent variable

The outcome variable was modern contraception use. In the ZDHS survey, participants were asked if they were *currently using any modern contraception*. Modern contraceptives include implants, injectables, male and female sterilisation, intrauterine devices (IUDs), contraceptive pills, patches, vaginal rings, female, and male condoms, lactational amenorrhea and vasectomy, as defined by the WHO [5]. The dependent variable was measured by the proportion of women who responded as ‘currently using one form of modern contraceptive’. The responses were dichotomised as either using modern contraception, yes = 1 or not using modern contraception, no = 0.

### Independent variables

Women’s Autonomy was the independent variable measured by 12 questions that described three dimensions of women’s autonomy: women’s participation in decision-making, women’s attitude towards wife beating and women’s household status. The questionnaire is validated by DHS Measure and other similar studies, and the components are not used in isolation, but as whole dimensions [11–14].

#### Women’s participation in decision-making

Respondents were asked about their involvement in decision-making in five areas: contraception use, health care, major household purchases, respondent’s earnings and visiting relatives. Decisions regarding contraception use and health care indicate women’s ability to make decisions regarding family planning and health, while decisions on significant household purchases indicate decision-making with a partner [7]. Independent decisions about visiting relatives, and spending one’s own earnings suggested influence by the partner over women’s social life and finances [8]. A score of one [1] was given if women participated in decision-making and zero [0] if they did not participate. A composite index was created with a scale from zero to five, with zero indicating no involvement and five indicating high participation in decision-making.

#### Women’s attitude towards wife-beating (domestic violence)

Women were also asked about their level of acceptance regarding wife or partner beating in any of the following situations: beating justified if she denies husband/partner sex, neglects children, goes out without informing husband/partner and burns food. A score of zero [0] was given if women responded yes to any of the questions and one if women said no. A composite index was created with a scale of 0 to 5, with zero [0] indicating wife /partner beating justified and five [5] indicating not justified [11–13].

#### Women’s household status

Women’s household status was measured by property ownership, such as land or house. Women were asked about ownership of major properties. A score of one [1] was given if women indicated property ownership and zero [0] if respondents did not own any property [7, 11–13].

Total scores measuring women’s autonomy are shown in Table 1. The final autonomy index was created from the summation of the three dimensions of women’s liberation, zero [0] to twelve [12] [Cronbach’s a = 0.80 (0.797)].

**Table 1:** Summary of measure of women’s autonomy

Questions	Possible outcomes	Score
1 Women’s Participation in Decision-Making		
Who makes the final decision Regarding?		
*a. Contraception use* *b. Health care* *c. Large Household purchases* *d. Respondent’s earnings* *e. Visit to relatives*	*Respondent alone*	*1*
	*Respondent and partner*	*1*
	*Respondent and other*	*1*
	*Husband or partner*	*0*
	*Someone else*	*0*
	*Other*	*0*
*Total score for participation in decision-making (A)* = *score(a)* + *score(b)* + *score(c)* + *score(d)* + *score(e)* *Maximum attainable score of 5*		
2 Women’s Attitude towards Wife Beating		
***Wife beating justified if:***		
*a. She denies husband/partner sex* *b. Neglect’s children* *c. Burns food* *d. Goes out without informing their husband/partner* *e. If argues with husband/partner*	*Yes*	*0*
	*No*	*1*
	*Do not know*	*0*
*Total score for women’s attitude towards wife beating (B) is score(a)* + *score(b)* + *score(c)* + *score(d)* + *score(e)* *A maximum attainable score of 5*		
3 Women’s Household Status		
*Woman:*		
*a. Owns land* *b. Owns a house*	*Does not own*	*0*
	*Owns alone*	*1*
	*Joint ownership*	*1*
	*Both alone and jointly*	*1*
*Total score for Women’s Household status (C) is score(a)* + *score(b)* *Maximum attainable score of 5*		
**The Overall score for women’s Autonomy is A + B + C** **The maximum attainable score of 12: A score of 0–4 = low autonomy, 4–8 = moderate autonomy and 8–12 = high autonomy**		

#### Control variables

Potential confounders such as women’s age, level of education, religion, region of residence, number of children, wealth index group and set up of residence were used as control variables. These were measured as follows: level of education (0 = no education; 1 = primary education; 2 = secondary education; 3 = higher education), household wealth quintiles (0 = poorest; 1 = poorer; 2 = middle; 3 = richer; 4 = richest), Urban or rural residence (0 = urban; 1 = rural), religious affiliations (0 = protestant, catholic = 1, Islam = 2 other-3), and number of children (zero = 1, one to three = 2, 4 and above = 3. The above indicators were chosen based on associations observed and validated in other studies [9, 11, 16, 17].

### Statistical analysis

Data analysis was conducted using Windows Statistics Package for Social Sciences version 25 (IBM-SPSS). Bivariate and multivariate analyses examined the relationships between independent and outcome variables. Bivariate associations were examined between dimensions of women’s autonomy, autonomy tertiles and modern contraception use. Multivariate logistic regressions were used to investigate the correlation between women’s autonomy and contraceptive use and other socio-demographic variables. The maximum likelihood estimates of odds ratio (OR) and 95% confidence interval (CI) adjusted for necessary variables were calculated.

## Results

The study included data for 6727 partnered women obtained from the 13 683 women in the 2018 ZDHS.

We found that 91% of women were not using modern contraceptives and 72% of the women had low autonomy levels. Women with moderate and high autonomy levels were more likely to use modern contraception than those with lower autonomy levels. Table 2 summarizes the association between contraceptive use and socio-demographic factors of partnered women in Zambia.

**Table 2:** Percentage of partnered women and crude odds ratio of modern contraceptive use and selected demographic variables (N = 672)

Variable	Using modern contraceptives			*P* value
	Frequency (%)	Crude odds ratio (OR)	95% confidence interval	
Age (mean = 32 years)				
15–24	22.6	2.021	1.653–2.475	0.003
25–34	37.5	1.063	1.019–1.831	0.000
35–49	39.8	Ref		
Education				
No education	10.5	Ref		
Primary education	52	0.228	0.144–0.363	0.000
Secondary	31.9	0.416	0.308–0.563	0.000
Higher	5.5	3.123	2.078–3.410	0.002
Setting of residence				
Urban	35	1.835	1.547–2.178	0.000
Rural	65	Ref		
Wealth index				
Poorest	22.9	0.306	0.232–0.403	0.000
Poorer	21.3	0.351	0.268–0.460	0.000
Middle	20.8	0.483	0.376–0.620	0.000
Richer	17.3	2.663	1.932–3.731	0.002
Richest	17.7	Ref		
Number of children				
0	3.2	Ref		
1–3	48.3	1.734	1.313–3.992	0.003
4 +	48.5	1.110	0.936–1.317	0.230
Women’s autonomy (tertiles)				
Low	71.9	Ref		
Moderate	19.5	1.690	1.160–2.462	0.001
High	8.5	1.414	1.263 -2.147	0.000

### Multivariate analysis

Table 3 illustrates the percentage of partnered women by dimensions of women’s autonomy. While Table 4 shows the results from the multivariate analysis. Women’s autonomy was significantly related to modern contraception use and other covariates used in the study, such as education, wealth index and age.

**Table 3:** Percentage of partnered women by dimensions of women’s autonomy (N = 6727)

Autonomy Variable	Frequency (%)	Using modern contraceptives		*P* value
		Crude odds ratio (OR)	95% CI	
Participation in decision making				
Scores				
0	5.1	1.168	1.047–1.303	0.005
1	8.3			
2	11.0			
3	16.9			
4	58.7			
Attitude towards wife beating				
0	16.6	1.080	1.032–1.130	0.001
1	9.3			
2	7.9			
3	8.2			
4	8.1			
5	50.0			
Woman’s household status				
0	40.8	0.808	0.732–0.891	0.000
1	22.0			
2	37.2			
Women’s autonomy (tertiles)				
Low	71.9	Ref		
Moderate	19.5	1.690	1.160–2.462	0.006
High	8.5	1.414	0.931–2.147	0.001

**Table 4:** Adjusted odds ratio for multivariable logistic regression models predicting modern contraceptive use by selected variable

Variable	Adjusted odds ratio	95% confidence interval	*P* value
Age			
15–24	1.266	1.182 -2.113	0.007
25–34	1.163	1.052–1.273	0.002
35–49	Ref		
Education			
No education	Ref		
Primary education	1.516	0.744–3.089	0.321
Secondary	1.073	0.666–1.728	0.360
Higher	1.181	1.091–1.783	0.012
Number of children			
0	2.041	1.102–3.780	0.037
1–3	2.221	1.257–3.924	0.001
4 +	Ref		
Wealth index			
Poorest	0.692	0.562–0.841	0.215
Poorer	0.391	0.037–0.960	0.290
Middle	1.840	1.147–2.952	0.018
Richer	1.230	1.105–1.766	0.006
Richest	Ref		
Setting of residence			
Urban	Ref		
Rural	0.838	0.592–0.914	0.014
Women’s autonomy			
Low	Ref		
Moderate	1.054	1.048–1.312	0.004
High	1.03	1.013–1.562	0.001

## Discussion

This study is the first to investigate the association between women’s autonomy and modern contraception use among partnered women in Zambia. Of the 6727 partnered women analysed in this study, only 8.8% of women were currently using a modern method of contraception. Women with moderate and high autonomy levels were significantly associated with modern contraception use, although over 70% had low levels of autonomy. Other factors related to increased modern contraception use included increased level of education, age and increased wealth. Our result differs from the 2018 ZDHS report, where modern contraception use was reported to be 43%. The difference between our findings and the ZDHS findings could be due to the differing variables used in the data analysis. Nevertheless, both results obtained are still lower than the set target of 50% as stipulated in the 2017 National Health Strategic Plan [3, 4].

Other studies evaluating women’s autonomy and modern contraception use have similarly used demographic health surveys to explore this question [10, 18–20]. Women with high or moderate autonomy were more likely to be assertive enough to make independent choices regarding modern contraception than those with low autonomy [15, 21, 22]. In reaching women’s autonomy and level of education, women’s autonomy could bear more influence on contraception use, as seen from a Pakistan study which found that women’s decision-making autonomy was more significantly associated with contraception use than education [16]. Other cultural factors, such as those that promote men as heads of relationships, may influence decision-making in women who are educated [23–25]. All these issues are essential indicators of gender equality and are women’s rights issues [26].

Formal education improves women's understanding of sexual and reproductive health, including modern contraception use and women's decision-making, as was found in another study in Zambia [10]. However, most women in Zambia still do not have formal education, which can partly explain the low contraception use among partnered women [19]. Age was another feature associated with contraception use and reflects findings reported in similar studies [20]. Like other sub-Saharan African countries, Zambia has a lower fertility age; the median age at first birth in Zambia is 19.2 among those aged between 20 and 49 [4]. Desire to delay pregnancy owing to younger age and to wanting a longer birth interval could be additional factors as to why modern contraception use was higher among younger women. Wealthy women were more likely to report modern contraception use than those in the poorest category; this result is similar to findings from studies undertaken in Ghana, which showed wealthy women had a higher likelihood of using modern contraceptives [21, 22, 31, 32]. The poverty level in Zambia stands at 88%, with about 60% of its population living below the poverty line of less than $1 US per day [19, 33, 34].

Based on the findings in this study, women’s autonomy was significantly associated with modern contraception use among partnered women in Zambia. Although we did not analyse wife-beating as an independent factor, other studies have found that women subjected to domestic violence or intimate partner violence are less likely to use contraception or access health care [35–37].

Our findings show that modern contraception use among partnered women in Zambia is low. This could explain the high maternal mortality rate due to unplanned pregnancies [4]. To achieve the sustainable development goals (SDG 3 and 5), programs and policies that encourage women to get involved in decision-making must be promoted. Women were empowered to take up leadership roles at all levels of society [1, 2, 5]. Women must be given some degree of independence in decision-making regarding sexual and reproductive health.

The findings of this study highlight the importance of gender equality in decision making by affirming the evidence finding that enhancing women’s autonomy improves the uptake of contraception use [24, 25].

Strategies to improve women's autonomy include improving women’s socioeconomic status, enhancing their education and dismantling of cultural laws that support gender inequality [14, 26]. Increasing the socio-economic status of women is one of the most powerful tools that impacts significantly on reproductive choice [26].

Women’s autonomy could also be improved by enhancing women's level of education [16]. This can improve self-awareness and enhance knowledge enabling women to learn more about self-value through interactions with others [27]. The Zambian government developed an education policy allowing pregnant girls to continue their education to reduce early marriages and retain girls in schools [28]. Nevertheless, more targeted policies that would promote already married women, particularly in rural areas, to have access to a basic level of education are required [29]. Male involvement, particularly in sexual and reproductive health, is another strategy that could enhance women's autonomy [9, 30, 38, 39]. It promotes confidence and enhances morale towards positive decisions, consequently improving the uptake of sexual reproductive health services such as contraception [30, 40, 41]. Traditional norms and cultural beliefs are barriers that inhibit women's autonomy [23]. Therefore, abolishing certain practices, such as patriarchal norms, that inhibit women's participation in households would empower women to participate in decision-making [23, 38, 39].

### Limitations and strengths of the study

Limitations of this study include the use of current women’s contraception use status, which may not reflect past and future contraception choices. In addition, autonomy was only measured using women’s responses without involving responses from their partners and made the assumption that all partners were male. Moreover, the study was cross-sectional, used secondary data for analysis, could only explore associations, and could not infer causality. This means that results must be interpreted with caution. A longitudinal study design may be able to better study the relationship. Despite the limitations, this study's strengths included using a large representative sample from a reputable source, the DHS, that uses sound sampling methods with a high response rate. In addition to this, previously validated measures were used in this study [31, 32].

## Conclusion

Our study is the first to investigate the relationship between women’s autonomy and modern contraception use among partnered women in Zambia. In HIC, the modernisation of society and the introduction of policies that enhance equality irrespective of sex, religious affiliations, gender and colour have created a community that enhances women’s autonomy [33, 34]. Therefore, adapting these policies to LMIC could be beneficial in strengthening women’s autonomy, thereby improving the uptake of sexual and reproductive health services. Thus, there is a need for continued efforts to roll out interventions to enhance women’s freedom to reduce gender inequality.

## Data Availability

The datasets generated during and analysed during the current study consent to participate available from the corresponding author on reasonable request from DHS-Measure.
